# Genome-wide association study using specific-locus amplified fragment sequencing identifies new genes influencing nitrogen use efficiency in rice landraces

**DOI:** 10.3389/fpls.2023.1126254

**Published:** 2023-07-14

**Authors:** Zuyu Liao, Xiuzhong Xia, Zongqiong Zhang, Baoxuan Nong, Hui Guo, Rui Feng, Can Chen, Faqian Xiong, Yongfu Qiu, Danting Li, Xinghai Yang

**Affiliations:** ^1^ College of Agriculture, Guangxi University, Nanning, China; ^2^ Guangxi Key Laboratory of Rice Genetics and Breeding, Rice Research Institute, Guangxi Academy of Agricultural Sciences, Nanning, China; ^3^ Sugarcane Research Institute, Guangxi Academy of Agricultural Sciences, Nanning, China

**Keywords:** rice, NUE, SLAF-seq, SNPs, GWAS, candidate genes

## Abstract

Nitrogen is essential for crop production. It is a critical macronutrient for plant growth and development. However, excessive application of nitrogen fertilizer is not only a waste of resources but also pollutes the environment. An effective approach to solving this problem is to breed rice varieties with high nitrogen use efficiency (NUE). In this study, we performed a genome-wide association study (GWAS) on 419 rice landraces using 208,993 single nucleotide polymorphisms (SNPs). With the mixed linear model (MLM) in the Tassel software, we identified 834 SNPs associated with root surface area (RSA), root length (RL), root branch number (RBN), root number (RN), plant dry weight (PDW), plant height (PH), root volume (RL), plant fresh weight (PFW), root fractal dimension (RFD), number of root nodes (NRN), and average root diameter (ARD), with a significant level of *p* < 2.39×10^–7^. In addition, we found 49 SNPs that were correlated with RL, RBN, RN, PDW, PH, PFW, RFD, and NRN using genome-wide efficient mixed-model association (GEMMA), with a significant level of *p* < 1×10^–6^. Additionally, the final results for eight traits associated with 193 significant SNPs by using multi-locus random-SNP-effect mixed linear model (mrMLM) model and 272 significant SNPs associated with 11 traits by using IIIVmrMLM. Within the linkage intervals of significantly associated SNP, we identified eight known related genes to NUE in rice, namely, *OsAMT2;3, OsGS1, OsNR2, OsNPF7.4, OsPTR9, OsNRT1.1B, OsNRT2.3*, and *OsNRT2.2*. According to the linkage disequilibrium (LD) decay value of this population, there were 75 candidate genes within the 150-kb regions upstream and downstream of the most significantly associated SNP (Chr5_29804690, Chr5_29956584, and Chr10_17540654). These candidate genes included 22 transposon genes, 25 expressed genes, and 28 putative functional genes. The expression levels of these candidate genes were measured by real-time quantitative PCR (RT-qPCR), and the expression levels of *LOC_Os05g51700* and *LOC_Os05g51710* in C347 were significantly lower than that in C117; the expression levels of *LOC_Os05g51740*, *LOC_Os05g51780*, *LOC_Os05g51960*, *LOC_Os05g51970*, and *LOC_Os10g33210* were significantly higher in C347 than C117. Among them, *LOC_Os10g33210* encodes a peptide transporter, and *LOC_Os05g51690* encodes a CCT domain protein and responds to NUE in rice. This study identified new loci related to NUE in rice, providing new genetic resources for the molecular breeding of rice landraces with high NUE.

## Introduction

Nitrogen is one of the macronutrients necessary for rice growth and development, and its contribution to crop yield can reach 40%-50% (Daniel et al., 2007; Sylvester-Bradley and Kindred, 2009). However, excessive nitrogen application can not only cause eutrophication and soil acidification but also increase the cost of agricultural production (Allen et al., 2004; Rodrigo, 2012).

Quantitative trait locus (QTLs) has been identified as controlling NUE due to its inherent complexity (Sheng et al., 2006; Qian et al., 2015). Based on this, many efforts have been made to improve NUE through agronomic practices or genetic dissection (Liu et al., 2023). Previous studies have demonstrated the potential of manipulating genes directly responsible for N uptake and assimilation to improve rice NUE (Gojon, 2017). For example, there has been much progress in QTLs related to nitrogen utilization, such as the discovery of *qNUE6* (Yang et al., 2017), *qRDWN6XB* (Anis et al., 2019), *and qRDW-6* (Anis et al., 2018) on rice chromosome 6. Similarly, the introduction of *indica OsNR2* into Nipponbare improved its effective tiller number, grain yield, and NUE (Gao et al., 2019). Increased *OsNRT2.1* transcription from the *OsNAR2.1* promoter led to significant increases in rice yields and NUE (Chen et al., 2016). It has been found that the overexpression of glutamine synthases *OsGS1.1* and *OsGS1.2* in rice reduces grain yield and disrupts C metabolism (Cai et al., 2009). The introgression of *OsTCP19*-H into two japonica cultivars significantly increased tiller, indicating the potential for improvement in rice NUE with *OsTCP19*-H (Liu et al., 2021). Furthermore, *OsDREB1C* overexpression promotes early flowering and increases NUE (Wei et al., 2022). The above evidence suggests that manipulating genes directly involved in nitrogen uptake and assimilation is a good way to improve NUE in rice.

Rice mainly absorbs nitrate and ammonium from the soil. NRT1s and NRT2s are the major nitrate transporters in rice. According to the affinity of nitrate transporters, NRTs can be divided into two categories: low-affinity and high-affinity. The NRT1/PTR family belongs to low-affinity nitrate transporters (**Table 1**). *OsNRT1* is the first identified low-affinity nitrate transporter in rice, which regulates nitrate uptake in roots (Lin et al., 2000). *OsNRT 1.1* has two splice forms: *OsNRT1.1a* and *OsNRT1.1b* which differ in their splicing patterns (Xiaorong et al., 2016). *OsNRT1.1A* can improve NUE and promote flowering (Wang et al., 2018). *NRT1.1B* affects the NUE of rice by regulating the rice root microbiome. Moreover, *NRT1.1B* not only has the function of nitrate uptake and transport but also has the function of sensing nitrate signals (Hu et al., 2015; Zhang et al., 2019). *OsNPF2.2* can export nitrate from the xylem and can also transport nitrate from root to stem, affecting the overall growth and development of the plant vascular system (Li et al., 2015). *OsNPF2.4* is mainly expressed in rice epidermis, xylem tissue, and phloem sieve tube companion cells, playing critical roles in NO_3_^-^ absorption, inter-organ transport, and redistribution (Xia et al., 2015). *OsNPF4.5* is specifically expressed in mycorrhizal arbuscules and participates in mycorrhizal symbiotic pathways for nitrate uptake and mycorrhizal formation (Wang et al., 2020). *OsNPF5.16* encodes a pH-dependent, low-affinity nitrate transporter that positively regulates rice tiller number and yield by modulating cytokinin levels (Wang et al., 2022). *OsNPF6.1* encodes a nitrate transporter with two haplotypes: OsNPF6.1^HapA^ and OsNPF6.1^HapB^. *OsNPF6.1* is induced by nitrate and is mainly expressed in rice lateral roots, root epidermal cells, and leaf nodes. Under low nitrogen conditions, the expression of *OsNPF6.1* is higher in NILs-OsNPF6.1^HapB^, and its transport activity is also higher (Tang et al., 2019). *OsNPF7.2* is mainly expressed in the elongation zone and mature zone of roots, especially in the sclerenchyma, cortex, and stele of roots. It controls tiller bud growth and root development by regulating cytokinin levels and cell cycles (Hu et al., 2016). Both spliced transcripts of *OsNPF7.7* affect nitrogen uptake and distribution, and they positively regulate rice tillering and NUE. Overexpression of *OsNPF7.7-1* can promote root nitrate influx and concentration, while overexpression of *OsNPF7.7- 2* promotes the influx and concentration of ammonium ions in the root system. *OsNPF7.7RNAi* and *osnpf7.7* showed increased amino acid content in the leaf sheath and decreased amino acid content in the leaves, thereby affecting nitrogen distribution and plant growth (Weiting et al., 2018). The overexpression of *OsNPF7.1* or *OsNPF7.4* can promote nitrate uptake. *OsNPF7.1* overexpression can moderately increase the nitrate and amino acid concentrations, which in turn increases seedling biomass and yield. However, excessive nitrate in *OsNPF7.4*-overexpression plants may lead to the accumulation of amino acids in leaf sheaths, thereby inhibiting seedling biomass; moreover, the reduced nitrate reutilization rate in seedlings also limits the accumulation of plant biomass (Weiting et al., 2019). The NRT2 family belongs to the high-affinity nitrate transporters, among which OsNRT2.4 is a dual-affinity nitrate transporter (Wei et al., 2018). *OsNRT2.1*, *OsNRT2.2*, and *OsNRT2.3a* are transcriptionally upregulated by nitrate supply; they need to interact with the chaperone OsNAR2.1 to uptake different concentrations of nitrates. On the contrary, OsNRT2.3b and OsNRT2.4 can still function in the absence of NAR2 (Yan et al., 2011; Tang et al., 2012). *OsNRT2.1* is involved in nitrate-dependent root elongation by regulating auxin transport to the root. The overexpression of *OsNRT2.1* promoted the effect of NO_3_^-^ treatment on root growth, which required the active polar transport of auxin (Misbah et al., 2019). OsNRT2.3a is responsible for nitrate loading in roots and nitrate transport to shoots, but it has no effect on nitrate uptake in roots (Tang et al., 2012). *OsNRT2.3b* is mainly expressed in the phloem of the stem, playing a role in pH and ion homeostasis; it can also cause membrane potential depolarization and cytoplasmic acidification under NO_3_^-^ supply conditions (Fan et al., 2016). The responsible genes for nitrate uptake in roots were clearly identified as *OsNRT1.1a, OsNRT1.1b, OsNRT1.1A, OsNNT1.1B*, and *OsNPF2.4*. On the other hand, *OsNPF 2.2, OsNRT2.3a*, and *OsNRT2.3b* were found to be responsible for nitrate transportation.

**Table 1:** Genes involved in N use in rice.

Gene name	Gene Locus	Function	Reference
OsNRT1	LOC_Os03g13274	Low-affinity nitrate transporter	(Lin et al., 2000)
OsNRT1.1A	LOC_Os08g05910	Improve NUE and promote flowering	(Wang et al., 2018)
NRT1.1B	LOC_Os10g40600	Nitrate uptake and transport,sensing nitrate signal	(Hu et al., 2015; Zhang et al., 2019).
OsNPF2.2	LOC_Os12g44100	Nitrate transport, transport nitrate from root to stem	(Li et al., 2015)
OsNPF2.4	LOC_Os03g48180	Low-affinity nitrate transporter	(Xia et al., 2015).
OsNPF4.5	LOC_Os01g54515	Nitrate uptake and promotes mycorrhizal formation	(Wang et al., 2020)
OsNPF5.16	LOC_Os01g65200	Low-affinity nitrate transporter	(Wang et al., 2022).
OsNPF6.1	LOC_Os01g01360	Nitrate transporter	(Tang et al., 2019)
OsNPF7.2	LOC_Os02g47090	Low-affinity nitrate transporter	(Hu et al., 2016)
OsNPF7.7	LOC_Os10g42870	Nitrate transporter	(Weiting et al., 2018)
OsNRT2.1	LOC_Os02g02170	High-affinity nitrate transporter	(Misbah et al., 2019)
OsNRT2.3a	LOC_Os01g50820	High-affinity nitrate transporter	(Tang et al., 2012)
OsNRT2.3b	LOC_Os01g50820	High-affinity nitrate transporter	(Fan et al., 2016).
OsNRT2.4	LOC_Os01g36720	Dual-affinity nitrate transporter	(Wei et al., 2018).
OsNAR2.1	LOC_Os02g38230	Chaperone protein of OsNRT2.1, OsNRT2.2, OsNRT2.3a	(Yan et al., 2011)
OsAMT1.1	LOC_Os04g43070	Low-affinity NH_4_^+^ transporter	(Yang et al., 2015)
OsAMT1.2	LOC_Os02g40730	NH_4_^+^ transporter	(Konishi and Ma, 2021)
OsAMT1.3	LOC_Os02g40710	NH_4_^+^ transporter	(Li et al., 2022).
OsGS1.2	LOC_Os02g50240	NH_4_^+^ assimilation	(Bao et al., 2014).
OsGS2	LOC_Os04g56400	NH_4_^+^ reassimilation	(Wallsgrove et al., 1987)
OsNADH-GOGAT2	LOC_Os05g48200	NH_4_^+^ assimilation	(Tamura et al., 2011).
OsNLP3	LOC_Os01g13540	Transcription factor	(Hu et al., 2019)
OsNAC42	LOC_Os09g32040	Transcription factor	(Tang et al., 2019).
OsGRF4	LOC_Os02g47280	Transcription factor	(Li et al., 2018)
DST	LOC_Os03g57240	Transcription factor	(Han et al., 2022)

While rice can absorb nitrate nitrogen, paddy soil is typically flooded for prolonged periods, resulting in high concentrations of ammonium nitrogen. Therefore, ammonium nitrogen is considered to be the main form of nitrogen absorbed by rice (Wang et al., 1993). The absorption of ammonium in rice requires ammonium transporters (**Table 1**). In rice, there are 12 hypothesized ammonium transporters that can be classified into five categories (Nicolaus et al., 2000; Cheng-Hsun and Yi-Fang, 2010): OsAMT1.1, OsAMT1.2, OsAMT1.3, OsAMT2.1, OsAMT2.2, OsAMT2.3, OsAMT3.1, OsAMT3.2, OsAMT3.3, and OsAMT4 (Sonoda et al., 2003a; Sonoda et al., 2003b; Suenaga et al., 2003). These ammonium transporters can provide a stable nitrogen source for rice, especially when the rice root is submerged in water for a long time. Nitrogen absorbed by rice requires transformation before it can be utilized. OsAMT1.1 is a low-affinity NH_4_^+^ transporter, and the OsAMT1.1-mediated NH_4_^+^ uptake and transport are not affected by intracellular or extracellular pH but are regulated by feedback from substrate accumulation (Yang et al., 2015). Under low ammonium conditions, both root growth and ammonium uptake are inhibited after the knockout of *OsAMT1.3* (Li et al., 2022). OsAMT1.1, OsAMT1.2, and OsAMT1.3 synergistically regulate ammonium uptake in rice under low nitrogen conditions. When ammonium supply is low, the single mutants have unaltered growth and nitrogen accumulation. In contrast, the *amt1.1:1.2* double mutants exhibit decreased stem growth and nitrogen content by 30%, while the *amt1.2:1.3* double mutant is not affected. The triple mutant has the most significant phenotype, with 59% inhibition of stem growth and a 72% decrease in nitrogen accumulation (Konishi and Ma, 2021). These results suggest that OsAMT1; 1, OsAMT1; 2, OsAMT1; 3, OsAMT2; 1, and OsAMT3; 1 are responsible for ammonium uptake in rice roots.

The NO_3_^-^and NH_4_^+^ absorbed by crop roots undergo a series of assimilations before being utilized. The majority of NO_3_^-^ is transported to different parts of plants for assimilation and utilization. In the cytoplasm, NO_3_^-^ is reduced to NO_2_^-^ by nitrate reductase (NR), and NO_2_^-^ in the cytoplasm is converted to NH_4_^+^ by nitrite reductase (NiR). Following this, NH_4_^+^ participates in the glutamate cycle, which comprises glutamine synthetase/glutamate synthase (GS/GOGAT). GS catalyzes the binding of NH_4_^+^ and glutamate to produce glutamine. GOGAT then utilizes glutamine and 2-ketoglutarate (2-OG), an intermediate of the tricarboxylic acid cycle, to generate two glutamate molecules (Masclaux-Daubresse et al., 2010). Rice possesses a GS1 family, with *OsGS1.2* playing an important role in NH_4_^+^ assimilation in the root (Bao et al., 2014). On the other hand, *OsGS2* is mainly expressed in the chloroplasts of leaves and plays a dominant role in the reassimilation of NH_4_^+^ released by photorespiration (Wallsgrove et al., 1987). The majority of the absorbed NH_4_^+^ is assimilated in the form of glutamate and glutamine and transported to the aerial part. When the external NH_4_^+^ concentration increases, the expression of *OsGS1.2* and *OsNADH-GOGAT2* in root epidermal cells and outer cortex cells increases significantly, thus rapidly assimilating NH_4_^+^, and the resulting glutamine and glutamate are transported to the aerial part (Tamura et al., 2011).

The processes of nitrogen uptake, transport, assimilation, and regulation involve complex gene regulatory networks. Many transcription factors have been identified to participate in the regulation process (**Table 1**), such as *NLP* (Wu et al., 2020), *NAC42* (Tang et al., 2019), *BTB* (Araus et al., 2016), and *GRF4* (Li et al., 2018). *OsNLP3* is a core transcription factor gene involved in nitrate signaling. It can translocate to the nucleus and initiate the transcription of NUE-related genes (Hu et al., 2019). OsNAC42 can activate *OsNPF6.1*, especially for *OsNPF6.1^HapB^*, while OsNAC42M can only transcriptionally activate *OsNPF6.1^HapB^*, indicating that *OsNPF6.1^HapB^* is more sensitive to OsNAC42 and its mutants (Tang et al., 2019). *GRF4* is a positive regulator of plant carbon-nitrogen metabolism. It can promote nitrogen uptake, assimilation, and transport, as well as photosynthesis, carbohydrate metabolism, and transport, thereby promoting plant growth and development (Li et al., 2018). Additionally, a study found that the expression of *OsNR1.2* was controlled by a zinc finger transcription factor called DROUGHT AND SALT TOLERANCE (DST) (Han et al., 2022).

To identify new genes related to NUE, this study used genome-wide association analysis to identify the loci that affected 11 traits of 419 rice landraces, including RSA, RL, and RBN. By using the software MLM model, we detected 834 significantly associated SNPs (*p* < 2.39×10^–7^), and using GEMMA, we identified 49 significantly associated SNPs (*p* < 1×10^–6^). Finally, RT-qPCR was used to validate the genes involved in NUE, providing additional genetic resources for the cultivation of new rice landraces with high nitrogen efficiency.

## Materials and methods

### Material planting

The experimental materials were collected from 419 rice landraces in Guangxi, including 330 *indica* rice, 78 *japonica* rice, and 11 other rice. Three biological replicates were set up for the NUE experiment, with 12 plants in each replicate. To break dormancy, the rice seeds were soaked in water for 24 hours at an ambient temperature of 28°C. Then, they were put on a damp cotton cloth at the same temperature for 24 hours to allow germination. The germinated seeds were sown in 96-well culture boxes and cultured with the normal nitrogen-level nutrient solution (1 mM NH_4_NO_3_). The nutrient solution was a thousand-fold dilution of Yoshida culture solution B (Yoshida, 1976). The seedlings were all placed in an incubator, with an ambient temperature of 28°C, humidity of 80%, light intensity of 40%, and light/dark cycle of 13/11 h. The seedlings were cultured for 20 d.

### Phenotyping

The phenotype parameters included plant height, plant fresh weight, plant dry weight, root length, root number, root branch number, root volume, number of root nodes, root fractal dimension, average root diameter, and root surface area. Plant height was measured from the base of the rice to the leaf tip. Plant dry weight was obtained by weighing the dried samples. The number of root nodes is the sum of the root number, root branch number, and the number of points at which the roots cross. The root fractal dimension is a direct indicator of root development. A higher root fractal dimension indicates a more developed root system, while a relatively small root fractal dimension suggests a weaker root meristem ability. The root data was obtained by scanning and analyzing the roots with the LA-S root scanning system (WSeen, Hangzhou, China).

### Specific-locus amplified fragment sequencing and SNP genotyping

Specific-locus amplified fragment sequencing was performed on an Illumina Hiseq 2500 system. The clean reads were clustered using BLAT software to obtain polymorphic SLAF tags. Then, the BWA software was used to align the polymorphic SLAF tag sequence to the Nipponbare reference genome (http://rice.uga.edu/). GATK and SAM toolkits were used to analyze SNP calling. A total of 208,993 SNPs were obtained based on a minor allele frequency (MAF) > 0.05 and a deletion rate < 0.5 (Yang et al., 2018).

### Genome-wide association analysis

The genetic relationship between samples was calculated using the Centcred_IBS module of Tassel. The population structure was analyzed by ADMIXTURE software. We conducted GWAS using the TASSEL software on 208,993 SNPs genotypes and seedling phenotype data. The MLM used a (Q+K) model, where Q was the population structure and K was the kinship coefficient. The SNPs with *p* < 2.39×10^–7^ were considered to have a significant association. Manhattan and Q-Q plots were generated in the R environment.

At the same time, GEMMA, another commonly used software for GWAS, was used to conduct association analysis on 208,993 SNPs genotypes and seedling phenotype data. The SNPs with *p* < 1×10^–6^ were considered to have significant correlations. Manhattan and Q-Q plots were also generated in the R environment.

### Candidate gene prediction

The Nipponbare was used as the reference sequences (http://rice.uga.edu/). The candidate regions were selected based on LD decay of 419 rice landraces, with 150-kb intervals upstream and downstream of the SNPs showing a significant correlation.

### Total RNA extraction and RT-qPCR

The materials for extracting RNA were the variety with the smaller RN: C117, and the variety with the larger RN: C347. The total RNA was extracted with an RNA extraction kit following kit instructions. For reverse transcription, 1 μg of RNA was used as the template for reverse transcription to synthesize cDNA. A 20 μl PCR reaction system was prepared following the kit instructions. The system consisted of 1X RT buff, 1 mM d NTPs, 0.5 μM oligo-dT primer, and 0.5 U RNase inhibitor. The 20 μl RT-qPCR system was prepared with 2xUniversal Blue SYBR Green qPCR Master Mix (Servicebio, Wuhan, China), plus 0.4 μl forward/reverse primers, 2 μl product cDNA, and 7.2 μl Nuclease-Free Water. Actin was used as the internal reference gene (Gaur et al., 2012). All primers used for RT-qPCR are in **Supplementary Table 1**

The RT-PCR reaction was carried out on the BIO-RAD T100 Thermal Cycler PCR instrument (Bio-Rad, California, USA), and the RT-qPCR reaction was carried out on the BIO-RAD CFX 96 Touch fluorescence quantitative PCR imaging system (Bio-Rad, California, USA).

### Statistical analysis

RT-qPCR data were analyzed using the 2^^ΔΔCt^ method (Thomas and Kenneth, 2008). Statistical analysis and plotting were performed using Origin 2022b and GraphPad Prism 8 software.

## Results

### Rice phenotypic analysis under normal nitrogen levels

The scope of research on NUE covers the entire process of nitrogen uptake, transport, assimilation, redistribution, and signal transduction in plants, which are essential for the improvement of NUE. In this study, 11 traits relating to N uptake, transport, and assimilation were investigated, and the genes associated with these traits were identified under unoptimized N conditions.

The seedlings of 419 rice landraces had significant differences in traits such as PH, PFW, and PDW after 20 days of hydroponic cultivation (**Table 2**). The data distribution of PH and RFD were less diverged, with a small coefficient of variation, indicating small genetic variations. The data distribution of PFW, PDW, RSA, ARD, and RL was more diverged and exhibited a higher divergence, indicating greater genetic variabilities. The data distribution of RBN, RN, NRN, and RL was the most divergent among all traits, with the largest coefficient of variation, showing a high degree of genetic variabilities.

**Table 2:** Phenotypic analysis of 11 traits in 419 rice landraces.

Traits	Average value	Standard deviation	Maximum	Minimum	Coefficient of variation (%)
PH	29.07	4.04	41.43	16.76	14%
PFW	0.15	0.03	0.45	0.07	23%
PDW	0.02	0.01	0.08	0.01	25%
RSA	7.64	2.40	28.87	3.71	31%
RBN	115.19	52.66	459.87	30.99	46%
RFD	1.35	0.04	1.57	1.26	3%
RN	214.90	112.14	998.92	55.77	52%
NRN	460.08	240.62	2209.37	116.52	52%
ARD	0.34	0.06	0.66	0.21	19%
RV	0.21	0.12	1.52	0.06	57%
RL	73.84	25.16	257.07	26.69	34%

**Figure 1:**
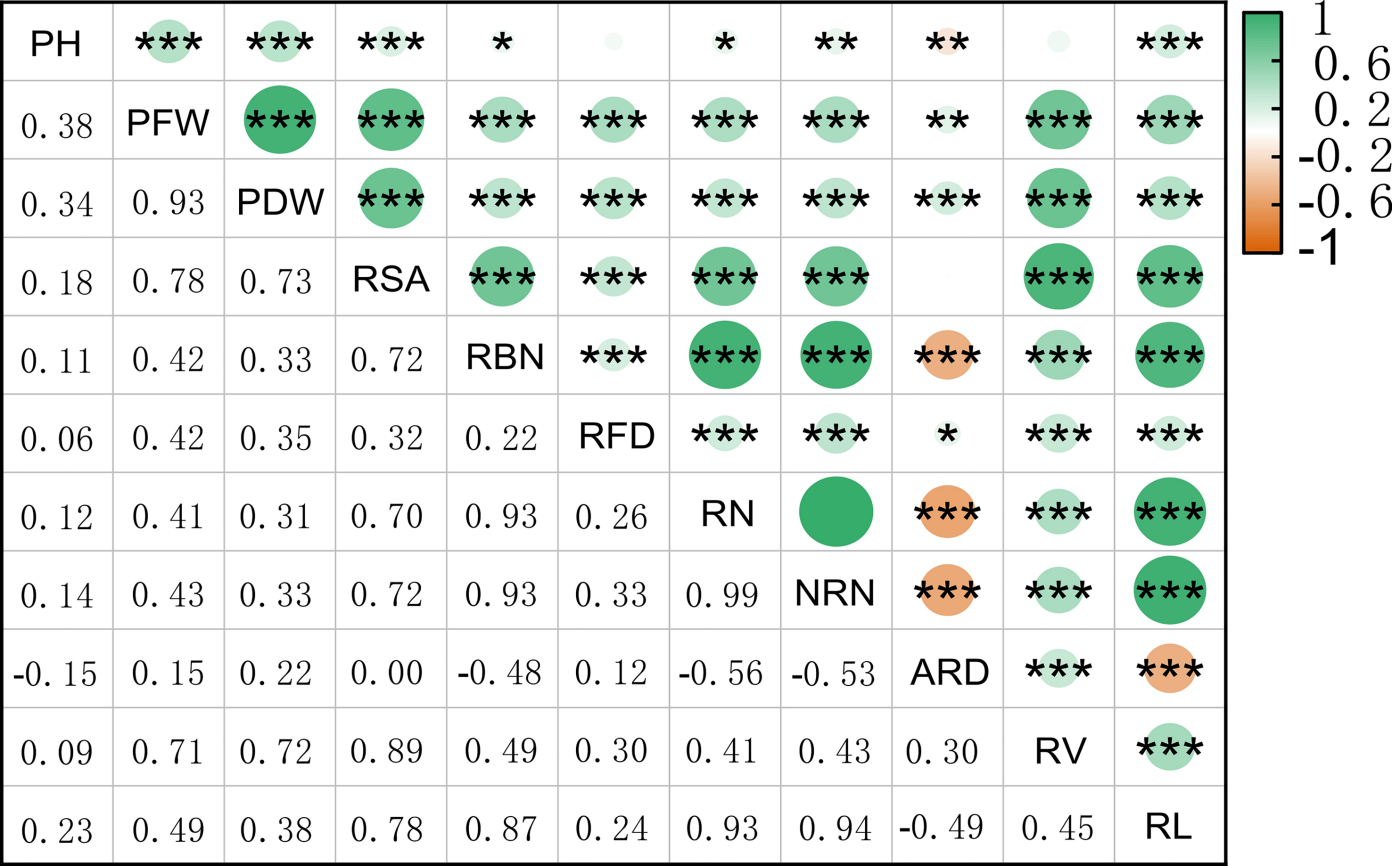
Pearson correlation coefficient plot for 11 rice traits. The color box in the upper left corner represents the correlation size.*p < 0.05,**p < 0.01, and ***p < 0.001.

**Figure 2:**
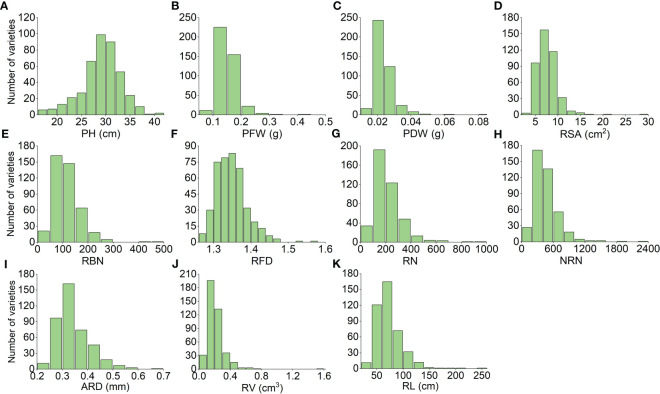
Frequency distribution map of 11 personality **(A–K)** traits of 419 rice landraces. The x-axis shows the quantitative value of the trait, and the y-axis shows the number of varieties that reached the value. The abscissa of RBN, RFD, RN, and NRN only indicates the magnitude of the numeric value.

We used Origin 2022b software to analyze the correlation among the 11 different traits and found that the PFW and PDW were highly positively correlated. Additionally, RSA and RV were also highly positively correlated; RBN was significantly positively correlated with RN, NRN, and RL, while NRN was significantly positively correlated with RBN and RL. On the other hand, ARD was significantly negatively correlated with PH, as well as multiple traits. Overall, the correlations among the root traits were significant (**Figure 1**). To reflect NUE, we identified PH PH, PFW, PDW, RSA, RBN, RFD, RN, NRN, ARD, RV, RL under normal nitrogen level (**Figure 2**). After 20 days of cultivation, 419 rice landraces showed differences in 11 traits.C51 has the largest PFW, RFD, RV, PDW and RSA. The C93 has the largest ARD. C339 has the largest PH. C349 has the largest RBN, RN, RL and NRN.

### Genome-wide association analysis

#### Phylogenetic tree construction and principal component analysis

The construction of the phylogenetic tree construction and principal component analysis for the 419 rice landraces were performed in the previous study (Yang et al., 2018).

#### Population structure and LD analysis

**Figure 3:**
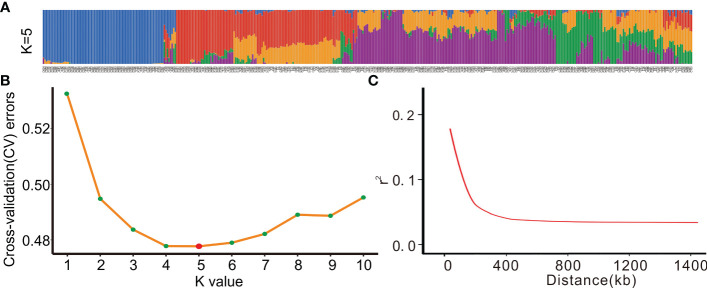
Population structure and LD decay value trend of 419 rice landrace.**(A)** Population distribution of 419 rice landrace at a K value of 5.**(B)** The cross-validation error rate (CV errors) can be used to determine the number of groups, and K value corresponds to a CV error, and the lower the CV errors, the more accurate the group.**(C)** Genome-wide average LD decay estimated from 419 rice landraces(r^2^).

To analyze the population structure of the entire population based on the screened SNPs, we used the admixture software and classified the samples (K value) into 1-10 groups. As a result, the 419 rice landraces were divided into five populations (**Figure 3A**), with the minimum cross-validation error rate (CV errors), indicating that all the samples might belong to these five populations (**Figure 3B**).

Next, genome-wide SNPs were used to analyze the LD level of the total population, and the physical distance corresponding to the 1/2 maximum correlation coefficient value (r^2^) was taken. The result showed that the LD decline distance of the total population on a genome-wide level was 150 kb (**Figure 3C**).

#### Genome-wide association analysis

**Figure 4:**
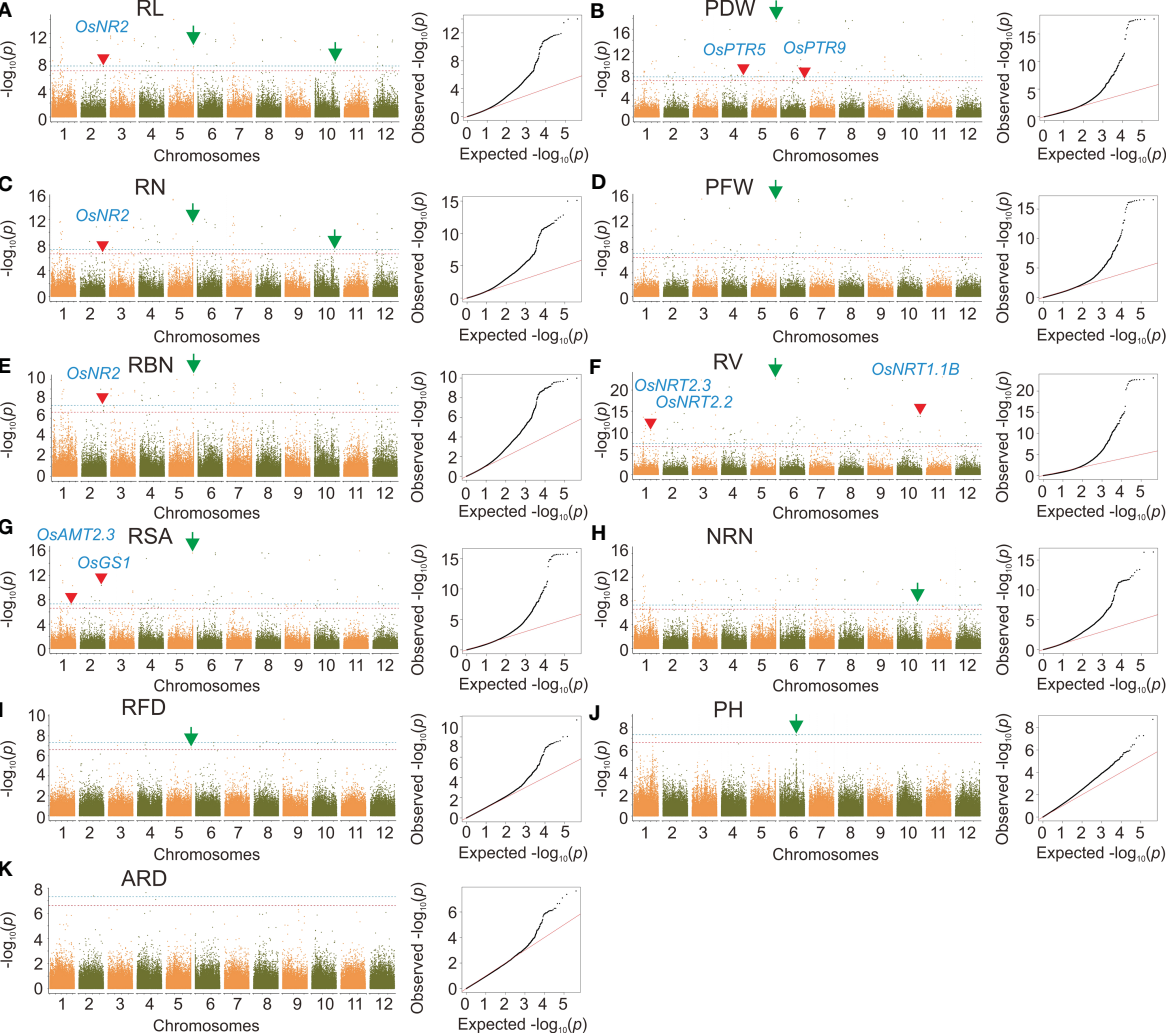
Genome-wide association analysis based on MLM. **(A-K)** Manhattan plots and quantile-quantile plots of the MLM model. Red arrows indicate significant sites associated with cloned nitrogen efficiency genes and green arrows indicate significant sites that can be further investigated. The red line indicates the significance threshold at *p* = 2.39×10^–7^. The blue line indicates the significance threshold at *p* = 4.78×10^–8^.

By using the MLM model, we identified 834 SNPs that were significantly correlated with RSA, RL, RBN, RN, PDW, PH, RL, PFW, RFD, NRN, and ARD, with a significant level of *p* < 2.39×10^–7^. These significantly associated SNPs were distributed on 12 rice chromosomes (**Supplementary Table 2** and **Figure 4**). Among all the traits, RV was associated with the most significant SNPs (157). In terms of the chromosomal distribution of significant SNPs, because PH and ARD had few significant SNPs, the significant SNPs of PH were only distributed on chromosomes 1 and 6, and the significant SNPs of ARD were distributed on chromosomes 2, 4, 9, and 10, while the significant SNPs of other traits were distributed on all 12 chromosomes, and the minimum number of the significant SNP on chromosome 1 was one, and the maximum was 22. For the loci effect, multiple traits shared the same significant SNPs. For example, Chr2_32491137 was associated with RL, RBN, and RN; Chr10_21705001 was associated with RSA, PDW RV, and PFW; Chr1_35486889 was associated with both RSA and RV. A peak SNP Chr5_29956584 was associated with RN, RL, and RBN (**Figures 4A, C, E**), and the most significant SNP Chr5_29804690 was also associated with RSA, RFD, PDW, PFW, and RV (**Figures 4B, D, F, G, I**). Chr10_17540654 was detected for RL, RN, and NRN (**Figures 4A, C, H**). In addition, a highly significant SNP Chr6_20714026 was identified for PH (**Figure 4J**).

**Figure 5:**
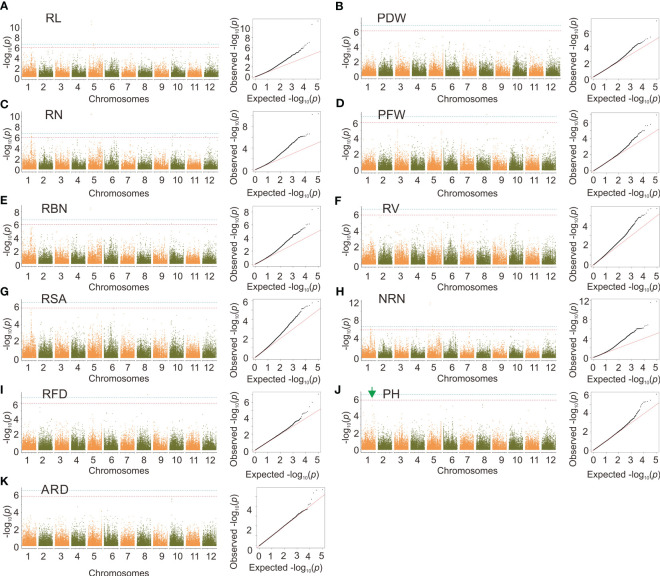
Genome-wide association analysis based on GEMMA. **(A-K)** manhattan plots and quantile-quantile plots of the GEMMA. Green arrows indicate significant sites that can be further investigated. The red line indicates the significance threshold at *p* = 1×10^–6^. The blue line indicates the significance threshold at *p* = 1×10^–7^.

We identified a total of 49 SNPs correlated with RL, RBN, RN, PDW, PH, PFW, RFD, and NRN using GEMMA, with a significant level of *p* < 1×10^–6^. These SNPs were distributed on all chromosomes other than 9 and 11 (**Supplementary Table 2** and **Figures 5 A–K**). We also identified that the same significant SNPs were detected in multiple traits. For example, Chr5_6117508 and Chr5_6117514 were identified in RL, RBN, RN, and NRN. The most significant SNP Chr1_32712980 was also identified for PH (**Figure 5J**).

We identified 272 final SNPs correlated with RSA, RL, RBN, RN, PDW, PH, RL, PFW, RFD, NRN, and ARD by using IIIVmrMLM (**Supplementary Table 2** and **Supplementary Figure 1**). We identified 193 final SNPs correlated with RL, RBN, RN, RSA, PH, PFW, RV, and NRN by using mrMLMN (**Supplementary Table 2** and **Supplementary Figure 2**).

Eight common SNPs were identified from both GEMMA and MLM, such as Chr5_6117508 and Chr5_6117514, which were associated with RL, RBN, RN, and NRN, as well as Chr1_32712980 for PH. There were eight SNPs in common between mrMLM and IIIVmrMLM, five SNPs in common between MLM and mrMLM, and nine SNPs in common between MLM and IIIVmrMLM. There were four SNPs in common between GEMMA and mrMLM and only one SNP in common between GEMMA and IIIVmrMLM.

#### Candidate gene analysis

According to the level of LD decay, candidate genes were selected within 150 kb upstream and downstream of significant SNPs. In the MLM model, the cloned NUE genes *OsAMT2.3* and *OsNRT2.3* (Gaur et al., 2012; Fan et al., 2016) were identified in the linkage intervals of SNP Chr1_35486889 and Chr1_29156411 on chromosome 1, which were significantly associated with RSA and RV. On chromosome 2, *OsGS1* was identified in the linkage intervals of Chr2_30716371, which was associated with RSA; *OsNR2* was identified in the linkage intervals of Chr2_32491137, which was associated with RL, RBN, RN, and PDW; *OsNRT2.2* was found in the linkage intervals of Chr2_691901, which was associated with RV (Feng et al., 2011; Lee et al., 2013; Gao et al., 2019). On chromosome 4, *OsNPF7.4* was identified in the linkage interval of Chr4_30171095, which was associated with RL, RBN, RN, and PDW (Leran et al., 2014). On chromosome 5, there were 14 candidate genes identified within the linkage interval of Chr5_29956584, which was associated with RN, RL, and RBN; in addition, there were 37 candidate genes in the linkage interval of Chr5_29804690, which was associated with RSA, RFD, PDW, PFW, and RV. On chromosome 6, *OsPTR9* was identified in the linkage interval of Chr6_29837500, which was associated with RL, RBN, RN, and PDW (Fang et al., 2013). In the linkage interval of Chr6_20714026, 47 candidate genes were identified, which were associated with PH. On chromosome 10, *OsNRT1.1B* was identified in the linkage interval of Chr10_21705001 (Hu et al., 2015), which was associated with RSA and RV. In the linkage interval of Chr10_17540654, 38 candidate genes were identified, which were associated with RL, RN, and NRN. In GEMMA, 41 candidate genes were identified within the linkage interval of Chr1_32712980, which was associated with PH.

Finally, based on the *p*-value of SNP and gene annotation, the candidate genes associated with Chr5_29956584, Chr5_29804690, and Chr10_17540654 were selected (**Supplementary PAGE \# "'Page: '#''" Q[CE] The emphases (colored text) from revisions were removed throughout the article. Confirm that this change is fine.Table 3**) because these SNPs appeared in multiple traits, and their candidate genes might have high research significance.

#### Expression analysis of candidate genes

In the MLM model, Chr5_29956584 was the most significant SNP associated with RN, RL, and RBN; Chr5_29804690 was the most significant SNP associated with RSA, RFD, PDW, PFW, and RV; Chr10_17540654 was associated with RL, RN, and NRN. These three significant SNPs appeared in multiple traits, and therefore the associated candidate genes associated with these SNPs were chosen for subsequent expression analysis. The Q-Q plot of MLM and GEMMA results showed that the model of RN was the best in both analysis methods, and thereby RNA was extracted from the materials of RN (**Figures 4**, **5**).

**Figure 6:**
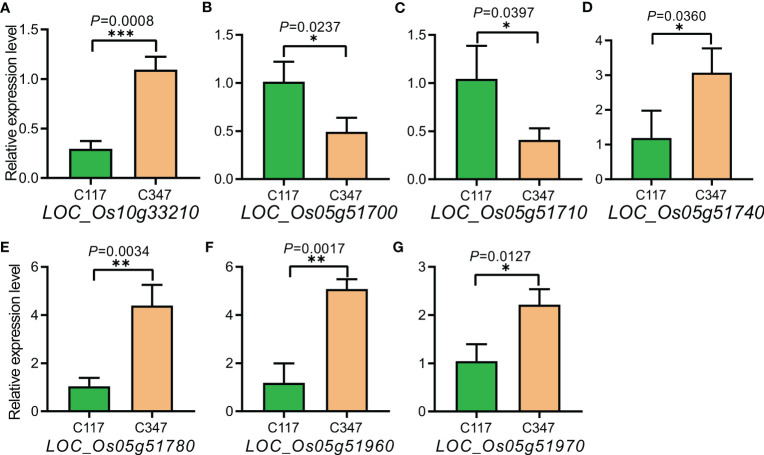
Expression analysis of candidate genes in C117 and C347. **(A-G)** The expression amounts of *LOC_Os10g33210*,*LOC_Os05g51700*,*LOC_ Os05g51710*,*LOC_Os05g51740*,*LOC_Os05g51780*,*LOC_Os05g51960* and *LOC_Os05g 51970* respectively. The x-axis represents the material used to detect the amount of gene expression, and the y-axis represents the amount of gene expression in the material. *p < 0.05, **p < 0.01, and ***p < 0.001.

Among 419 landraces, we selected the varieties with differences in both RSA and RN to measure 22 important genes in the linkage intervals of Chr5_29804690, Chr5_29956584, and Chr10_17540654 using RT-qPCR (**Supplementary Figures 3**, **4**). The results showed that *LOC_Os05g51720*, *LOC_Os05g51820*, *LOC_Os05g51860*, and *LOC_Os05g51900* had little or no expression in C117 or C347. The expression levels of *LOC_Os05g51690*, *LOC_Os05g51750*, *LOC_Os05g51754*, *LOC_Os05g51790*, *LOC_Os05g51800*, *LOC_Os05g51810*, *LOC_Os05g51830*, *LOC_Os05g51850*, *LOC_Os05g51870*, *LOC_Os05g52080*, and *LOC_Os05g52090* were not significantly different between C117 and C347 (**Supplementary Figures 5A-K**). The expression levels of *LOC_Os05g51700* and *LOC_Os05g51710* were significantly lower in C347 than that in C117, and the expression levels of *LOC_Os05g51740*, *LOC_Os05g51780*, *LOC_Os05g51960*, *LOC_Os05g51970*, and *LOC_Os10g33210* were significantly higher in C347 than that in C117 (**Figures 6A–G**).

## Discussion

In recent years, GWAS has become an effective technology to detect complex trait loci in rice. In this study, we used the MLM model to detect 834 SNPs significantly associated with 11 traits, and we used the GEMMA model to identify 49 SNPs significantly associated with eight traits. We identified 272 final SNPs by using IIIVmrMLM, and 193 final SNPs by using mrMLMN. Due to the difference in SNP screening criteria and the *p*-value threshold between the Tassel and GEMMA software, the number of detected association SNPs was also different.

### Association of nitrogen utilization of known genes with studies

Among the candidate genes associated with significant SNP from the MLM model, eight were known NUE-related genes, including *OsAMT2.3*, *OsGS1*, *OsNR2*, *OsNPF7.4*, *OsPTR9*, *OsNRT1.1B*, *OsNRT2.3*, and *OsNRT2.2*. *OsAMT2.3* encodes an ammonium transporter protein. When nitrogen input is increased, the number of grains per panicle, thousand-grain weight, percentage of nitrogen in biomass, and protein content in grains between nitrogen-efficient and nitrogen-inefficient varieties show significant differences; the gene expression of *OsAMT2.3* was also different in the flag leaves at the filling stage (Gaur et al., 2012). In this study, *OsAMT2.3*-associated SNPs were detected in RSA and RV, suggesting that *OsAMT2.3* may also affect these two parameters. *OsGS1* encodes a cytoplasmic glutamine synthetase. *OsGS1* is expressed in all rice tissues and is highly expressed in leaves. When rice uses ammonium as the main nitrogen source, *OsGS1* plays an important role in coordinating the entire metabolic network and can affect the normal growth and filling of rice (Tabuchi et al., 2005; Kusano et al., 2011). *OsGS1*-associated SNPs were detected in RSA. *OsGS1* is also expressed in roots and is important for root growth. *OsNR2* encodes nitrate reductase, which exhibits a high enzyme activity and is known for its sensitivity to hypochlorite as well as its ability to absorb high amounts of nitrate. *OsNR2* promotes the expression of *OsNRT1.1B* in indica rice 9311, which in turn enhances the expression of *OsNRT1.1B* (Gao et al., 2019). *OsNR2*-associated SNPs were detected in RL, RBN, and RN. *OsNR2* is expressed in the vascular tissue of rice shoots and roots, as well as the elongation zone of young roots, but *OsNRT1.1B* was not found in the linkage intervals of the SNP significantly associated with these three traits. It is possible that the interaction between *OsNR2* and *OsNRT1.1B* was not obvious in the rice landraces included in this study. *OsNPF7.4* encodes a nitrate transporter. Knockout *OsNPF7.4* can increase seedling biomass, tiller number, seed number, and yield per plant, and excessive nitrate in plants with high *OsNPF7.4* expression may lead to amino acid accumulation in leaf sheaths, thereby inhibiting the seedling biomass (Weiting et al., 2019). *OsNPF7.4*-associated SNPs were detected in PDW. Since the high expression of *OsNPF7.4* expression could inhibit seedling biomass, it may also affect seedling PDW. *OsPTR9* encodes a peptide transporter that localizes to the plasma membrane. The expression of *OsPTR9* is regulated by exogenous nitrogen and circadian rhythm. The overexpression of *OsPTR9* can increase ammonium uptake, promote the formation of lateral roots, and increase yield; the downregulation of *OsPTR9* has the opposite phenotypic effect (Fang et al., 2013). *OsPTR9*-associated SNPs were detected in PDW. The positive effect of *OsPTR9* on lateral root formation may increase PDW. *OsNRT1.1B* encodes a nitrate transporter, which is mainly expressed in root hairs, epidermis, and vascular tissues, and it is also highly expressed in epidermal cells and stele cells adjacent to the xylem in roots. *OsNRT1.1B* affects the NUE of rice by regulating the microorganisms with nitrogen transformation ability in roots (Hu et al., 2015; Zhang et al., 2019). *OsNRT1.1B*-associated SNPs were detected in RSA, PDW, RV, and PFW. Since *OsNRT1.1B* is expressed in roots and regulates root microbiome, root surface area and volume can affect the contact area between roots and microorganisms; thereby, *OsNRT1.1B* may be involved in the regulation of RSA and RV. *OsNRT2.2* encodes a high-affinity nitrate transporter, which is up-regulated by nitrate and inhibited by NH_4_^+^ and high temperature; its expression level is increased by light or exogenous sugar treatment (Feng et al., 2011). *OsNRT2.3* is localized on the plasma membrane and is mainly expressed in the parenchyma cells of the root xylem. It is responsible for nitrate loading in the root and transport to the aerial part and has no effect on the uptake of nitrate in the root (Tang et al., 2012). Both *OsNRT2.2* and *OsNRT2.3*-associated SNPs were detected in RV, so both *OsNRT2.2* and *OsNRT2.3* may have a certain effect on RV.

### Association of candidate genes with studies

The key to plant development is the uptake of micronutrients and macronutrients by the root system. The use of these resources, which are often unevenly distributed in the soil, is optimized as root architecture responds to nutrient availability (Kemo et al., 2017; Hans et al., 2019). Previous GWAS studies have successfully elucidated the adaptive mechanism of root structure to nutrients (Bouain et al., 2018). In a previous study, 96 varieties of *Arabidopsis thaliana* were screened under nitrogen treatment, and the GWAS results on seven root traits showed that only one-third of the genes were associated with the same trait (average lateral root length) under two different nitrogen concentrations (Miriam et al., 2017). In this study, we analyzed the RSA, RL, RBN, RN, PDW, PH, RV, PFW, RFD, NRN, and ARD of 419 rice landraces in a nutrient solution. The phenotypic data showed a large genetic diversity and a large correlation between different traits. Through gene expression analysis, we found that the expression of *LOC_Os05g51700* and *LOC_Os05g51710* in C347 was significantly lower than that in C117, while the expression of *LOC_Os05g51740*, *LOC_Os05g51780*, *LOC_05g51960*, *LOC_Os05g51970*, and *LOC_Os10g33210* in C347 was significantly higher than that in C117. Since *LOC_Os10g33210* was found in RL, NRN, and RN, it is likely to participate in the regulation of these traits. Although there are expression differences among *LOC_Os05g51700*, *LOC_Os05g51710*, *LOC_Os05g51960*, and *LOC_Os05g51970*, the functions of the encoded proteins are unknown, and they belong to hypothetical proteins. *LOC_Os10g33210* has been identified before (Jie et al., 2010; Leran et al., 2014). An intuitive tool called CRISPR-adapted Functional Redundancy Checker has been proposed to facilitate functional genomics in rice (Hong et al., 2020). *LOC _ Os10g33210* was found to have protein sequences and expression patterns similar to several nitrate transporter proteins (https://cafri-rice.khu.ac.kr/inspector). *LOC_Os05g51780* encodes a zinc finger protein, which belongs to a family of zinc finger transcription factors that can transmit signals. Some studies have found that zinc finger transcription factors are involved in nitrogen assimilation; under a condition with sufficient water, zinc finger transcription factors could up-regulate target genes *OsNR1.2* and *OsPrx24*, which can improve nitrogen assimilation and promote the opening of stomata. Under osmotic stress, zinc finger transcription factors could down-regulate the expression of *OsNR1.2* and *OsPrx24*, leading to inhibited nitrogen assimilation and stomatal closure, which enhances the drought tolerance of rice. *LOC_Os05g51780* was also associated with RSA, RFD, PDW, PFW, and RV. In *LOC_Os05g51690* and *LOC_Os05g51830*, although there was no significant difference in the expression of these two genes in C117 and C347, they were associated with RSA, RFD, PDW, PFW, and RV. *LOC_Os05g51690* is involved in the response to the lack of macronutrients, and it affects rice growth. Through alternative splicing, *LOC_Os05g51690* produces two transcripts with the same 5’ end, *NRRa* and *NRRb*, which encode 308aa and 223aa proteins, respectively. NRRa has one more CCT domain at the C-terminus than NRRb. *NRRa* and *NRRb* can regulate the structure of rice roots to improve macronutrient absorption; they also play a negative regulatory role in root growth and regulate the heading timing of rice (Yu-Man et al., 2012; Yuman et al., 2013). *LOC_Os05g51830* is located in the nucleus and expressed throughout the life cycle of rice. It can regulate rice seed germination under abiotic stress conditions. The overexpression of *LOC_Os05g51830* reduced the responses to abscisic acid (ABA), salt stress, and osmotic stress during seed germination and delayed seed germination (Zhao et al., 2014).

### Differences present in the studies

When analyzing the group structure, we divided the 419 rice landraces into five groups, while other studies divided the materials into six groups. The reason for the different groupings may be that the criteria used to filter SNPs were different. The GWAS results from the MLM model and GEMMA model were obviously different, and the MLM model generated much more significant SNPs than the GEMMA model. The different results of the two models may be caused by the strict SNP filtering in the GEMMA model. More than 200,000 SNPs were used in the MLM model, while less than 50,000 SNPs were used in the GEMMA model. On the other hand, the alleles associated with phenotypic diversity might have occurred in a low frequency, which made them hard to be detected by GWAS (Korte and Farlow, 2013).

## Conclusion

In this study, we used 208,993 SNPs to perform GWAS on 419 rice landraces. With the MLM model of the Tassel software, we detected 834 SNPs associated with RSA, RL, RBN, RN, PDW, PH, RV, PFW, RFD, NRN, and ARD under *p* < 2.39×10^–7^ significant levels, and they were distributed on 12 chromosomes. With GEMMA, we detected 49 SNPs associated with RL, RBN, RN, PDW, PH, PFW, RFD, and NRN under *p* < 1×10^–6^ significant level, and they were distributed on 10 chromosomes except chromosomes 9 and 11. RT-qPCR was used to detect the expression levels of candidate genes. The expressions levels of *LOC_Os05g51700* and *LOC_Os05g51710* in C347 were significantly lower than that in C117, while the expression levels of *LOC_Os05g51740*, *LOC_Os05g51780*, *LOC_Os05g51960*, *LOC_Os05g51970*, and *LOC_Os10g33210* were significantly higher in C347 than in C117. Comprehensive analysis indicated that *LOC_Os10g33210* and *LOC_Os05g51690* might be important candidate genes affecting NUE in rice. This study provides a theoretical basis for the genetic improvement of NUE in rice.

## Data availability statement

The datasets presented in this study can be found in online repositories. The names of the repository/repositories and accession number(s) can be found below: https://www.ncbi.nlm.nih.gov/, SRR6890917; https://www.ncbi.nlm.nih.gov/, SRR6890918; https://www.ncbi.nlm.nih.gov/, SRR6890919; https://www.ncbi.nlm.nih.gov/, SRR6891335.

## Author contributions

XY acquired the funding and participated in supervision; DL participated in supervision; ZL and YQ conducted the field trials and data collection and data analysis; ZL and XX carried out data visualization; BN and ZZ were in charge of the investigation; ZL, XY, and DL wrote, reviewed, and edited the draft. All authors contributed to the article and approved the submitted version.
