# Supplementary material for: Genome-wide association study using specific-locus amplified fragment sequencing identifies new genes influencing nitrogen use efficiency in rice landraces
Source: Front Plant Sci. 2023 Jul 14;14:1126254. doi: 10.3389/fpls.2023.1126254 (PMC10375723; doi:10.3389/fpls.2023.1126254)
Supplement: Supplementary file 1 [file DataSheet_1.docx]

**Figure S1**

Figure S1.Genome-wide association analysis based on IIIVmrMLMMLM. **(A-K)**Manhattan plots and quantile-quantile plots of the IIIVmrMLM model.

**Figure S2**

Figure S2.Genome-wide association analysis based on mrMLM. **(A-H)**Manhattan plots and quantile-quantile plots of the mrMLM model.

**Figure S3**

Figure S3.Landraces for extracting RNA

**Figure S4**

Figure S4.Analysis of the RSA and RN in rice varieties. **p* < 0.05, ***p* < 0.01, and ****p* < 0.001.

**Figure S5**

Figure S5.Expression analysis of candidate genes in C117 and C347.**(A-K)**The expression amounts of *LOC_Os05g51690*, *LOC_Os05g51750*, *LOC_ Os05g51754*, *LOC_Os05g51790*, *LOC_Os05g51800*, *LOC_Os05g51810* *LOC_Os05g51830, LOC_Os05g51850, LOC_Os05g51870, LOC_Os05g52080* and *LOC_Os05g52090* respectively.The x-axis represents the material used to detect the amount of gene expression, and the y-axis represents the amount of gene expression in the material.**p* < 0.05, ***p* < 0.01, and ****p* < 0.001.
