# Supplementary figures and images for: Genome-wide association study using specific-locus amplified fragment sequencing identifies new genes influencing nitrogen use efficiency in rice landraces
Source: Front Plant Sci. 2023 Jul 14;14:1126254. doi: 10.3389/fpls.2023.1126254 (PMC10375723; doi:10.3389/fpls.2023.1126254)

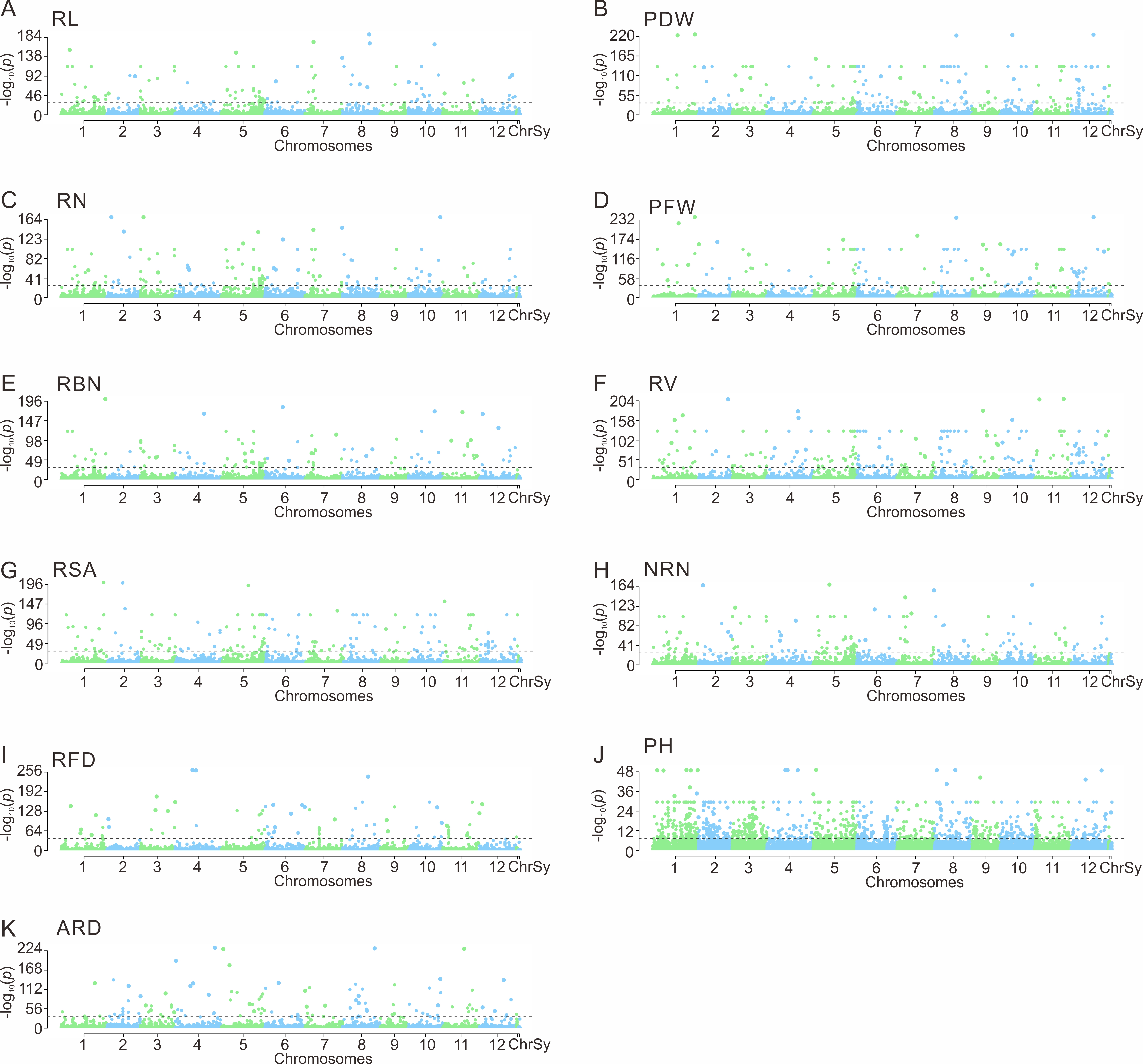

Supplement: Supplementary file 5 [file Image_1.jpeg]

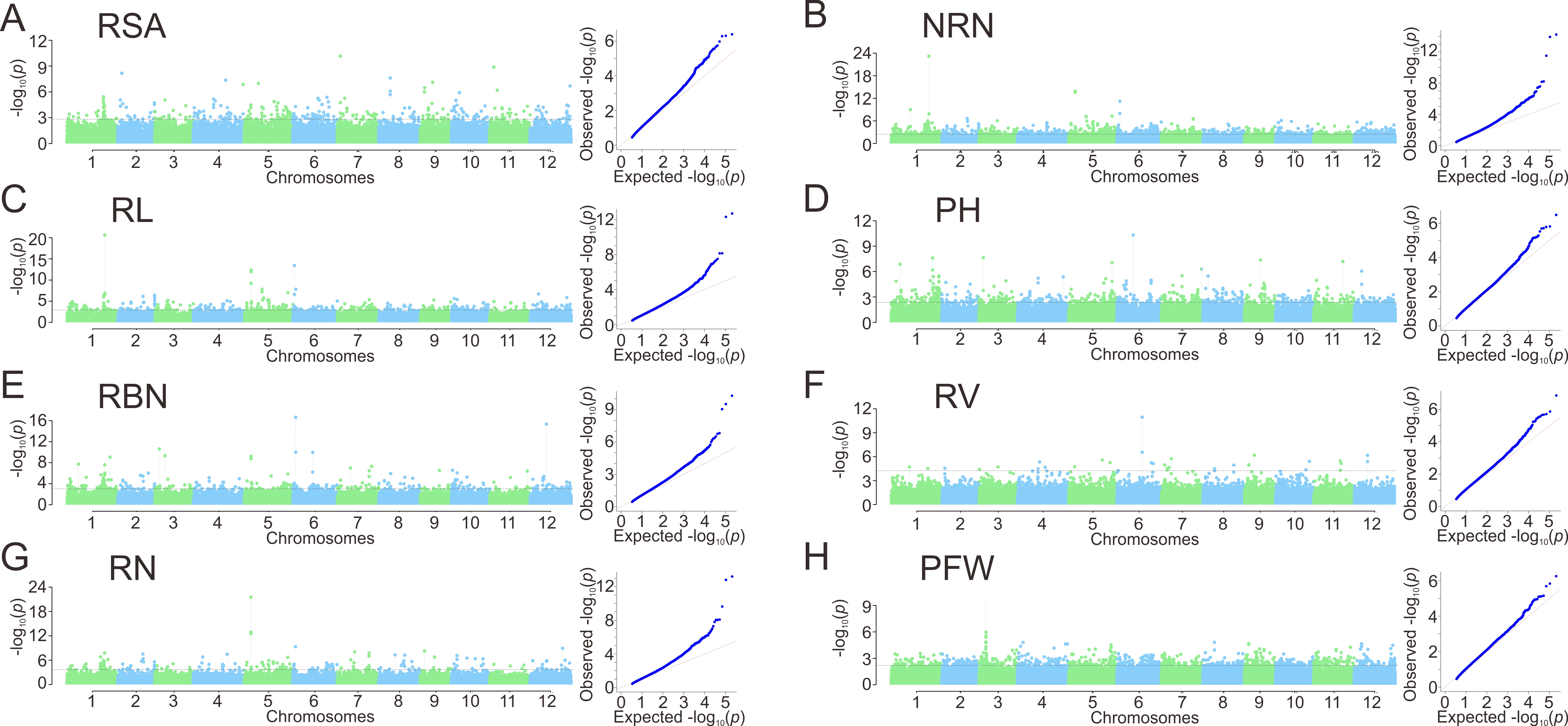

Supplement: Supplementary file 6 [file Image_2.jpeg]

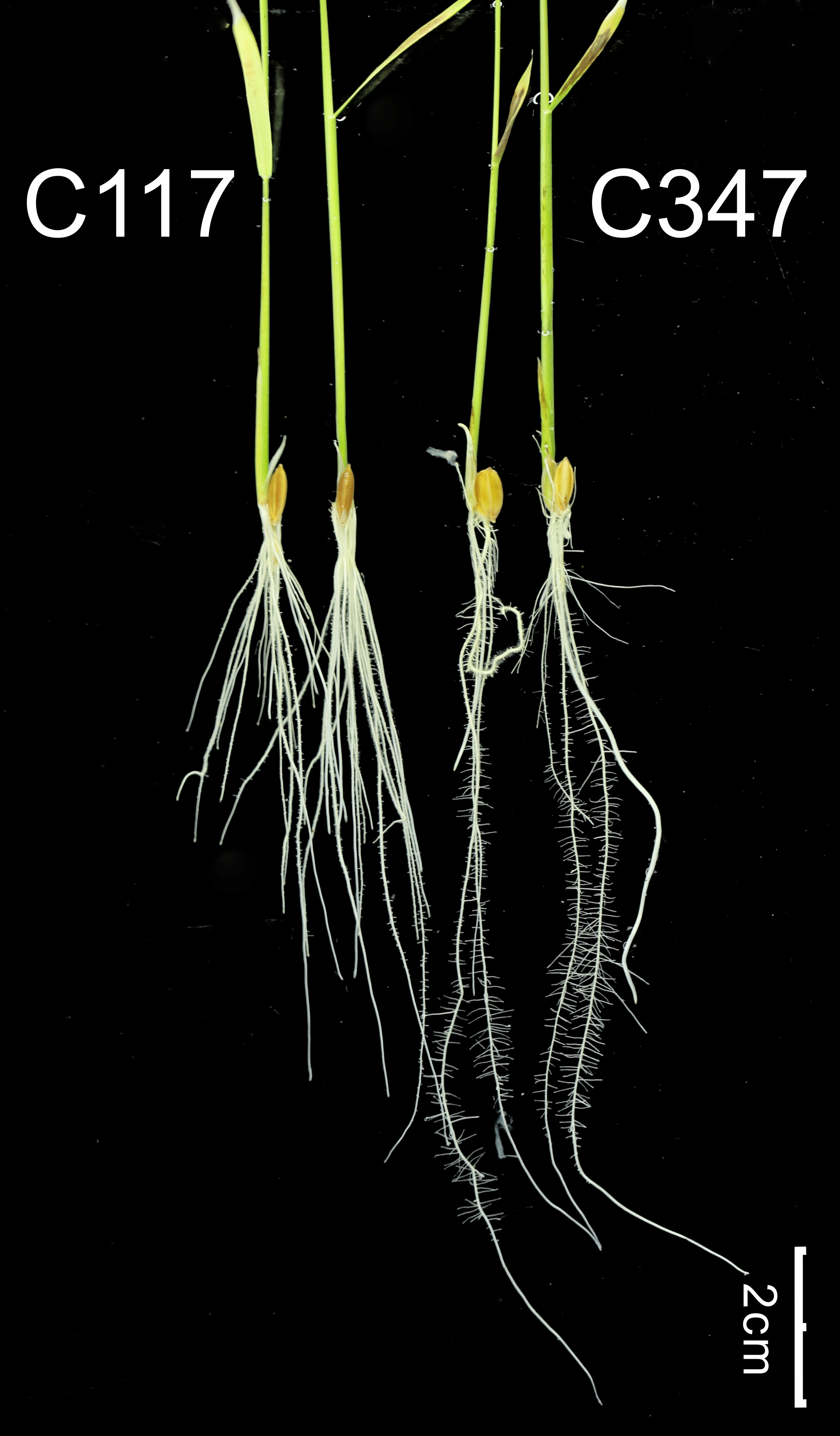

Supplement: Supplementary file 7 [file Image_3.jpeg]

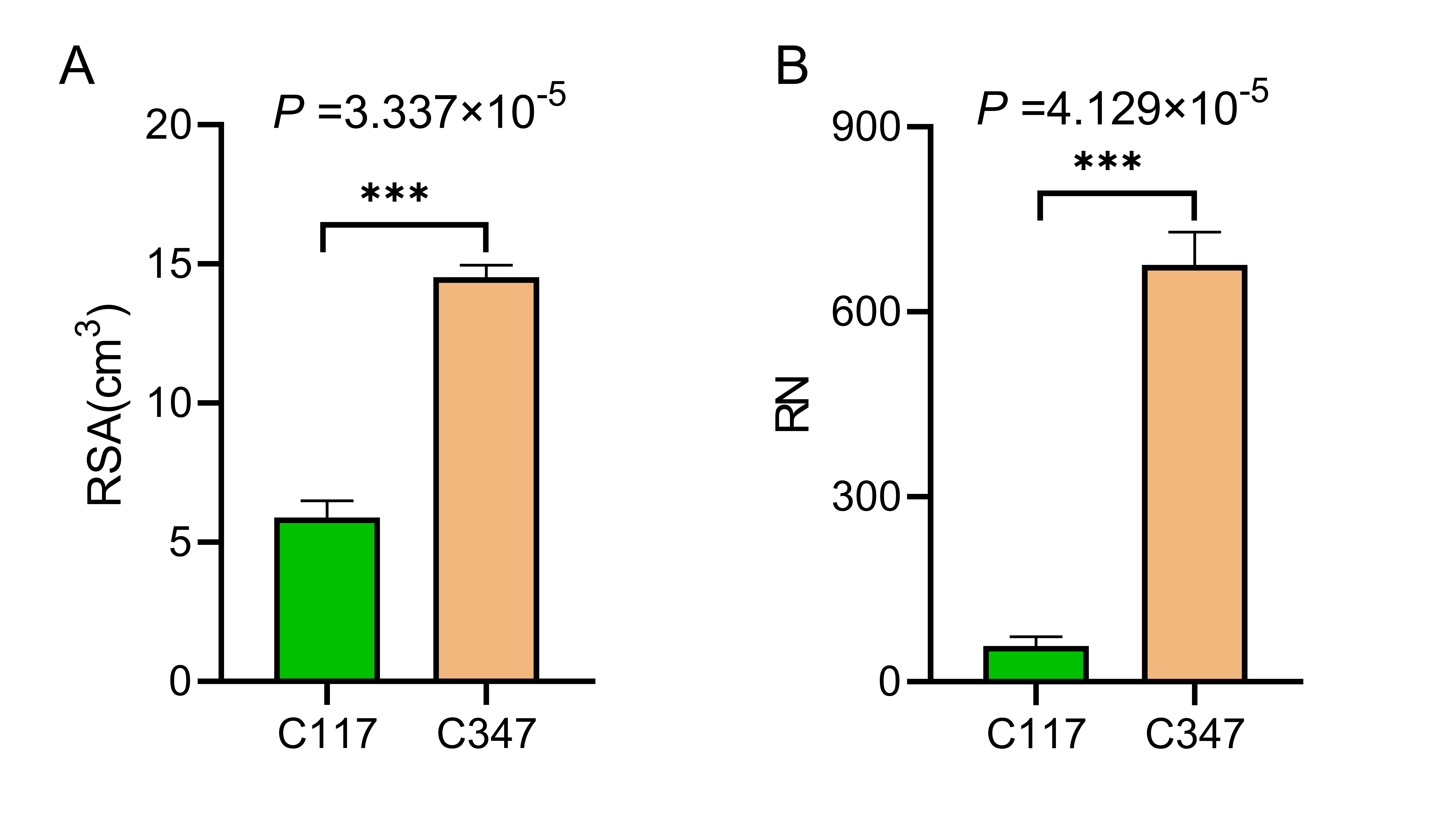

Supplement: Supplementary file 8 [file Image_4.jpeg]
